# Study of Complete Genome Sequence of Uncultivated *Hyphomonadaceae* sp.

**DOI:** 10.1128/mra.00765-22

**Published:** 2022-09-27

**Authors:** Xhesida Ajvazi, Lucas Serra Moncadas, Adrian-Stefan Andrei

**Affiliations:** a Faculty of Science, University of Zurich, Zurich, Switzerland; b Microbial Evogenomics Lab, Limnological Station, Department of Plant and Microbial Biology, University of Zurich, Kilchberg, Switzerland; University of Delaware

## Abstract

We report here a complete metagenome-assembled genome sequence belonging to the *Proteobacteria* phylum within the *Hyphomonadaceae* family. The circular chromosome was obtained from a metagenomic long-read sequencing run and displays a length of 4.1 Mbp along with a GC content of 62.5% at a coverage of 81×.

## ANNOUNCEMENT

*Hyphomonadaceae* members, usually found in oligotrophic marine habitats, exhibit optimized mechanisms for nutrients uptake. Despite their characterization in previous studies, *Hyphomonadaceae* family members cultivation remains challenging due to complex nutrient and growth factor requirements (1).

Unidentified Amoebozoa was non-axenically cultured from a Lake Zürich (47° 13' 20.9994"N, 8° 45' 9.72"E, Swiss Confederation) sample. The cultures were maintained in tissue culture flasks filled with 50 mL of autoclaved Swiss Alpina water (Pearlwater, Termen, Switzerland). Planktothrix rubescens strain A7 served as food source. DNA was extracted using the MagAttract HMW DNA kit (Qiagen, Hilden, Germany). It was further purified with Beckman Coulter AMPure XP magnetic beads and subsequently used for metagenomic sequencing on a Nanopore PromethION platform using a FLO-PRO002 (R9.4.1) flow cell. The 1D library was constructed with the SQK-LSK110 Ligation Sequencing kit (ONT, Oxford, UK) in conformity with manufacturer's instructions without any prior DNA fragmentation or size selection.

The obtained reads (approx. 2 million reads; mean read length 9 kbp, median read quality 11.7, N50 14.4 kbp) were basecalled and quality trimmed (Q score 7) with Guppy 5.1.15 prior to assembly by Flye 2.9-b1768 (2). Circularity of the genome was determined by evaluating the existence of repeated sequences at the end of the contig by using Flye software. Contamination of the metagenome-assembled genome (4 180 036 bp, coverage of 81×) was assessed by CheckM v1.1.3 (97.32% complete, 1.38% contaminated) (3). Taxonomical classification was performed with GTDB-Tk v1.4.0 (4) and by comparing its 16S rRNA, predicted by barrnap 0.9, against SILVA database v138 (5). Prokka v1.12 (6) and NCBI’s PGAP pipeline were used for gene prediction and functional annotation. The assignment of KO identifiers to orthologous genes was performed by BlastKOALA (7). Pathways reconstruction was accomplished with online KEGG Mapper-Reconstruction tool using the obtained KO numbers. PFAM domains were predicted using the pfam_scan.pl script with the PFAM database release 32 (8). gRodon R package (9) was applied to predict growth rates and defense mechanisms diversity was explored with DefenseFinder (10). Default parameters were used except where otherwise specified.

GTDB-tk classified the obtained genome as belonging to family *Hyphomonadaceae* (*Proteobacteria*), while 16S rRNA sequence showed 91.54% identity to *Aquidulcibacter paucihalophilus* strain TH1-2. With a GC of 62.5%, the recovered genome exhibited 4 246 CDS, 70 encoded transporters and 1 rRNA operon. Genome-inferred metabolic reconstruction suggested a gram-negative bacterium with slow duplication time (approx. 6.5 h) and an aerobic heterotrophic lifestyle. The acquired genome (strain JAD_PAG50586_4) had the metabolic potential of synthesizing all proteinogenic amino acids (except Alanine, Methionine) and to degrade 18 of them. A large spectrum of catabolic (i.e., TCA cycle, pyruvate oxidation, Entner-Doudoroff, and fatty acid degradation) and anabolic (i.e., gluconeogenesis and fatty acid biosynthesis) pathways were found complete. Interestingly, the chromosome displayed only two different defense mechanisms. All genes needed for flagellum assembly were found present pointing toward motility.

### Data availability.

All sequence data is available through the National Center for Biotechnology Information (NCBI) via BioProject PRJNA824509 (CP096134; GCA_023242925.1; SRX14779367). Additional proteome annotations (KEGG, Prokka, and Pfam) are available in figshare repository: https://doi.org/10.6084/m9.figshare.20375574.
